# Molecular evidence for trichomonads and acanthamoebae in cloacal samples of synanthropic waterfowl

**DOI:** 10.1007/s00436-025-08522-z

**Published:** 2025-07-02

**Authors:** Sándor Hornok, Andor Pitó, Sándor Szekeres, Nóra Takács, Krisztina Bárdos, Gergő Keve, Yuanzhi Wang, László Ózsvári

**Affiliations:** 1 Department of Parasitology and Zoology, University of Veterinary Medicine, Budapest, Hungary; 2 HUN-REN-UVMB Climate Change: New Blood-Sucking Parasites and Vector-Borne Pathogens Research Group, Budapest, Hungary; 3 Department of Veterinary Forensics and Economics, University of Veterinary Medicine, Budapest, Hungary; 4 National Laboratory of Infectious Animal Diseases, Antimicrobial Resistance, Veterinary Public Health and Food Chain Safety, University of Veterinary Medicine Budapest, Budapest, Hungary; 5 Key Laboratory for Prevention and Control of Emerging Infectious Diseases and Public Health Security, the XPCC, School of Medicine, Shihezi University, Shihezi, Xinjiang, Uygur Autonomous Region, China

**Keywords:** Mallard, Mute Swan, *Histomonas*, *Tetratrichomonas gallinarum*, *Simplicimonas*, *Acanthamoeba castellanii*, T4

## Abstract

Wild living birds that are infected with protozoan parasites may pose a risk of transmitting these to domestic fowl. While avian trichomonads are relatively often studied in this context, infection with acanthamoebae was only reported in a few terrestrial bird species. Taken together, the epidemiological role of wetland-associated bird species has been infrequently examined simultaneously for the presence of both groups of the above protozoan parasites. This study was initiated in the southern part of Central Europe, Hungary, to assess the role of waterfowl as carriers of trichomonads and acanthamoebae, involving 189 birds of 21 species from five avian orders sampled across three seasons (autumn, winter, spring). From these birds, cloacal swabs were collected and analyzed with molecular methods. *Tetratrichomonas gallinarum* (represented by three genetic variants) was detected in two synanthropic bird species, the Mallard (*Anas platyrhynchos*) and the Mute Swan (*Cygnus olor*), as well as a new *Tetratrichomonas* sp. in Ruffs (*Calidris pugnax*) and a *Simplicimonas* sp. in a Mallard. In addition, five *Acanthamoeba* spp., representing genogroups T4, T2, and T13, were demonstrated from Mute Swans and a Mallard exclusively in the autumn. Identical genotypes of trichomonads were only found in the same host species, even in multiple cases, while all five detected *Acanthamoeba* variants were represented by a single genotype per host, all of which occurred at the same location. These molecular data suggest that synanthropic waterfowl may harbor a broad spectrum of hind gut trichomonads and acanthamoebae, some of which may contaminate the environment and can be potentially acquired by domestic poultry and other vertebrates living nearby. Findings of the present study support that natural water surfaces and swimming may also entail infection with non-thermophilic opportunistic amoebae, and waterfowl should be added to the potential sources of such cases. This is particularly relevant to places where pastures are flooded with lake water.

**Supplementary Information:**

The online version contains supplementary material available at 10.1007/s00436-025-08522-z.

## Introduction

Birds play a significant epidemiological role in the transmission of pathogens, owing to their migration habits and presence in the surroundings of humans and domestic animals (Qiu et al. 2025). Synanthropic birds are especially important as carriers of parasites, some of which have a high veterinary-medical impact (Dipineto et al. 2013). Waterfowl may disperse mechanically a broad range of infectious agents, toward both humans and livestock, poultry (Elmberg et al. 2017). For instance, among protozoan parasites, the contribution of herbivorous wetland birds to outbreaks of cryptosporidiosis among grazing animals has long been recognized, because these birds frequently feed on pastures and can disperse oocysts acquired earlier from contaminated water (Wang et al. 2021). However, few studies addressed the potential role of waterfowl as carriers of flagellated protozoa or amoebae that might have a significant role as animal and/or human pathogens.

Trichomonads (Metamonada: Parabasalia: Trichomonadea) are flagellated protozoan parasites associated with the gut and genital mucosal surfaces of animals and humans. Apart from *Trichomonas gallinae*, *Tetratrichomonas gallinarum* is probably the most frequently diagnosed species in a broad range of wild living bird species, in which it is also considered potentially pathogenic, especially if it affects the liver with granulomatous necrosis (Cepicka et al. 2006). Infections caused by this and closely related species are commonly observed in geese. Most cases are subclinical, and the clinical form of the disease manifests with significant mortality and the presence of caseous contents in ceca (Falkowski et al. 2022). Lethal infection caused by *T. gallinarum* was also reported in Black Swans (*Cygnus atratus*) (Feng et al. 2021). The most optimal conditions for the survival and waterborne transmission of trophozoites are present in the aquatic environment; therefore, *T. gallinarum* mostly affects waterfowl such as anseriform birds (Cepicka et al. 2006; Feng et al. 2021). However, free-ranging domestic chickens and partridges (“land-associated” galliform birds) are also at risk of developing clinical signs and lesions due to infection with this flagellate (Liebhart et al. 2014; Landman et al. 2019). *Histomonas meleagridis*, on the other hand, is more often regarded as a pathogen of gallinaceous birds (Mitra et al. 2018), as it can survive outside the avian host while enclosed in worm eggs or paratenic hosts, but waterfowl such as ducks also appear to be susceptible to the infection and its pathologic consequences (Callait-Cardinal et al. 2006).

In addition to trichomonads, a few species of probably non-pathogenic amoebae (Amoebozoa: Discosea: Entamoebidae) are also known to infect galliform and anseriform birds, as exemplified by *Entamoeba gallinarum* and *Entamoeba anatis* (Levine 1985). At the same time, potentially highly pathogenic free-living, opportunistic amoebae are also present in the environment of birds (both in soil and water), as exemplified by acanthamoebae (Amoebozoa: Discosea: Acanthamoebidae) that can cause severe, even fatal encephalitis and keratitis in humans and other high vertebrates (Wang et al. 2023). On the other hand, reports are scarce on *Acanthamoeba*-infection among bird species. When their presence was documented in avian hosts, this involved unanimously non-aquatic birds, as exemplified by diurnal birds of prey (Karakavuk et al. 2017). Increasing the importance of this genus, highly pathogenic bacteria, such as *Escherichia coli*, *Mycobacterium* spp., and *Pseudomonas aeruginosa*, were observed in clinical isolates of acanthamoebae, whereas *Legionella*, adenovirus, mimivirus, and various undescribed bacteria (*Candidatus*) were often identified in environmental samples of the genus *Acanthamoeba* (Rayamajhee et al. 2021).

Thus, in light of the above, the aim of this study was to screen cloacal samples of wild living waterfowl with molecular methods for trichomonads and acanthamoebae, and thus to contribute to our knowledge on the epidemiological role of water-associated birds in veterinary and even medical contexts.

## Materials and methods

### Sample collection

During this study, cloacal swab samples were collected from 189 wetland birds representing 21 species of five orders (Table 1). English bird species names were capitalized below in the text following international recommendations (https://bou.org.uk/britishlist/bird-names/) (Gill et al. 2024). The samples were collected during bird ringing activities between August 23, 2023, and June 20, 2024, at 30 locations in Hungary, focusing on the northwestern part of the country (Supplementary Fig. 1). Water-associated birds were caught with either mist nets (Ecotone, Gdynia, Poland: 12 m in length, 2.5 m in height, with 16 × 16 mm holes) or funnel traps (Busse 2000). Cloaca swab samples (FLOQSwabs: Copan, Brescia, Italy) were taken by inserting the sterile cotton applicator into the cloaca 1–2 cm deeply, with circular movements then stored frozen at –20 °C.

**Table 1:** Sample sources and results of molecular identification of trichomonads and acanthamoebae in waterfowl. The three sampling periods were as follows: (1) autumn of 2023, from August 23 to November 03; (2) winter of 2023-2024, from December 8 to February 24; (3) spring of 2024 from March 17 to June 20. Bird names are used according to Gill et al. 2024.

Avian order	Bird species		Number of sampled birds: autumn-winter-spring	Results of molecular analyses	
	English name	Latin name		Number of positives for trichomonads: autumn-winter-spring (%)	Number of positives for acanthamoebae: autumn-winter-spring (%)
Pelecaniformes	Little Bittern	*Botaurus minutus*	2-0-1	-	-
	Grey Heron	*Ardea cinerea*	0–1-0	-	-
Charadriiformes	Northern Lapwing	*Vanellus vanellus*	0-0-4	-	-
	Black-headed Gull	*Chroicocephalus ridibundus*	1–22-31	-	-
	Green Sandpiper	*Tringa ochropus*	0-0-1	-	-
	Little Ringed Plover	*Charadrius dubius*	0-0-1	-	-
	Ruff	*Calidris pugnax*	0-0-15	0-0-7 (47%)	-
	Wood Sandpiper	*Tringa glareola*	0-0-18	-	-
	Common Snipe	*Gallinago gallinago*	0-0-4	-	-
	Mediterranean Gull	*Ichthyaetus melanocephalus*	0-0-6	-	-
	Curlew Sandpiper	*Calidris ferruginea*	2-0-0	-	-
	Common Gull	*Larus canus*	0–30-0	-	-
Gruiformes	Corn Crake	*Crex crex*	0-0-1	-	-
	Common Moorhen	*Gallinula chloropus*	2-1-0	-	-
Podicipediformes	Great Crested Grebe	*Podiceps cristatus*	2-0-0	-	-
Anseriformes	Mute Swan	*Cygnus olor*	6–7-8	1 (17%)−1 (14%)−0	4 (67%)−0-0
	Greylag Goose	*Anser anser*	0-0-3	-	-
	Garganey	*Spatula querquedula*	0-0-1	-	-
	Ferruginous Duck	*Aythya nyroca*	0-0-1	-	-
	Mallard	*Anas platyrhynchos*	3–10-3	0–4 (40%)−0	1 (33%)−0-0
	Red-crested Pochard	*Netta rufina*	0-0-2	-	-

### DNA extraction

DNA was extracted using the QIAamp® Fast DNA Stool Mini Kit and QIAamp® DNA Mini Kit (QIAGEN, Hilden, Germany) according to the manufacturer’s instructions with some modifications. Cloacal swabs were individually submerged into 2 ml sterile Sarstedt tubes containing 1 ml InhibitEx Buffer from the Fast Stool Kit. Thereafter, it was vortexed continuously for 1 min to get a thoroughly homogenized solution. The suspension was heated for 5 min at 70 °C, then vortexed for 15 s, and centrifuged for 1 min at 14,000 rpm (rotation per minute). The swab was carefully removed from the solution and drained as much as possible. Two hundred microliters of the supernatant was pipetted to a 1.5-ml microcentrifuge tube and 20 µl Proteinase K was added; then, the solution was thoroughly mixed by pulse-vortexing. The Proteinase K and further reagents were used from the DNA Mini Kit. Before pipetting 200 µl of Buffer AL, the solution was incubated at 56 °C for 1 h. Further steps were performed according to the standard DNA extraction protocol. DNA was eluted in 130 µl Buffer AE and stored at − 20 °C until molecular analyses.

### Conventional PCR analyses

For the detection of parabasalids (including the genera *Tetratrichomonas*, *Simplicimonas*, *Dientamoeba* and *Histomonas*), PCR amplification of a 603-bp-long part of the 18S rRNA gene was performed with the primers 18S-F (forward: 5′-GCA GTT AAA ACG CTC GTA GTC-3′) and 18S-R (reverse: 5′-AAC GCT AGA CAG GTC AAC CC-3′) (Bilic et al. 2014). The PCR was performed with the following conditions: an initial denaturation step at 95 °C for 5 min was followed by 40 cycles of denaturation at 95 °C for 30 s, annealing at 53 °C for 40 s, and extension at 72 °C for 1 min. In addition, a 480-bp-long part of the 18S rRNA gene of *Acanthamoeba* spp. was targeted with the primers JDP1 (5′-GGC CCA GAT CGT TTA CCG TGA A-3′) and JDP2 (5′-TCT CAC AAG CTG CTA GGG GAG TCA-3′) (Schroeder et al. 2001). Reaction conditions of this PCR included an initial denaturation at 95 °C for 5 min, followed by 35 cycles of denaturation at 95 °C for 35 s, annealing at 56 °C for 45 s, and extension at 72 °C for 1 min). Final extensions were performed at 72 °C for 10 or 7 min, respectively.

For each PCR method, 5 µl of extracted DNA was added to 20 µl of reaction mixture containing 1.0 U HotStar Taq Plus DNA Polymerase (5 U/µl) (Qiagen, Hilden, Germany), 0.5 µl dNTP Mix (10 mM), 0.5 µl of each primer (50 µM), 2.5 µl of 10 × Coral Load PCR buffer (15 mM MgCl_2_ included), and 15.8 µl distilled water. The *Acanthamoeba* spp. PCR mix contained 1 µl extra MgCl_2_ (25 mM) and less amount (14.8 µl) of distilled water.

### Phylogenetic and statistical analyses

The purification and sequencing of the PCR products were done by Eurofins Biomi Ltd. (Gödöllő, Hungary). The sequences obtained were manually edited using the BioEdit program and then compared to GenBank data by using BLASTn (https://blast.ncbi.nlm.nih.gov). The sequences obtained were submitted to GenBank (PV032157-PV032166). All sequences retrieved from GenBank had 99–100% coverage with sequences from this study and were trimmed to the same length prior to phylogenetic analysis. This dataset was resampled 1000 times to generate bootstrap values. Phylogenetic analysis was conducted with the neighbor-joining method, p-distance model using MEGA 11 (Tamura et al. 2021).

Fisher’s exact test was used to compare the rate of positive and negative samples between different sample categories, and differences were regarded significant if *P* < 0.05.

## Results

**Fig. 1:**
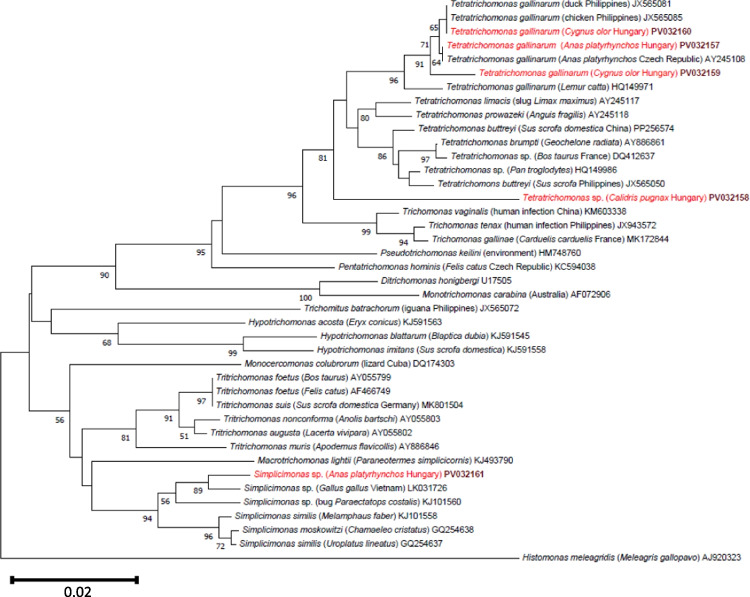
Phylogenetic tree of trichomonads based on the 18S rRNA gene. Evolutionary history was inferred using the neighbor-joining method. The percentage of replicate trees in which the associated taxa clustered together in the bootstrap test (1000 replicates) is shown below the branches. The tree is drawn to scale, with branch lengths in the same units as those of the evolutionary distances used to infer the phylogenetic tree. The evolutionary distances were computed using the p-distance method and are in the units of the number of base differences per site. In each row, after the species or genus name, the isolation source and the country of origin (if reported) are shown in parentheses, followed by the GenBank accession number. Sequences from Hungary are indicated with red fonts and maroon, bold accession numbers. The analyses involved 41 sequences and 544 positions. Evolutionary analyses were conducted in MEGA11

From 30 locations, 189 birds were sampled, which represented 21 species of five avian orders (Table 1). All birds were negative for *H. meleagridis*. However, other trichomonads were shown to be present in cloacal swabs of three species, the Ruff (*Calidris pugnax*), the Mute Swan (*Cygnus olor*), and the Mallard (*Anas platyrhynchos*) (Table 1). In 47% of examined Ruffs (7 of 15), the same *Tetratrichomonas* sp. was detected (GenBank: PV032158), which had a maximum of only 96% (504/525 bp) 18S rRNA gene sequence identity to *Tetratrichomonas* sp. from cattle (DQ412637) and 95.8% (503/525 bp) sequence identity to *Tetratrichomonas buttreyi* from pig (Germany: MK801500). Two closely related sequence variants of *T. gallinarum* were demonstrated from three Mute Swans (PV032159-PV032160, which were 99.05% (520/525 bp) identical to each other and the closest match in GenBank, from a duck sampled in the Philippines (JX565081). A further isolate from Mallard (*n* = 3, PV032157) was 99.8% (526/527 bp) identical to the latter sequence, and 99.8% (524/525 bp) to one of the conspecific sequences from Mute Swans (PV032160). In addition, a *Simplicimonas* sequence was successfully amplified from a single Mallard (PV032161). This genetic variant had the closest 100% (489/489 bp) sequence identity to *Simplicimonas* sp. reported from Austria (HG008105, excluded from the phylogenetic analysis because of low coverage) and 99.8% (513/514 bp) to a conspecific isolate from Vietnam (LK031726), both from chicken. While *Tetratrichomonas* sp. positive Ruff samples were collected in the spring, Mute Swans carried *T. gallinarum* during the autumn and winter, and PCR-positive Mallards were only found in the latter of the three sampling periods. The phylogenetic relationships of these avian trichomonads confirmed that they represented three species: *T. gallinarum* from Mute Swans and Mallard, a *Simplicimonas* sp. from the latter host species, and a probably new *Tetratrichomonas* sp. from Ruffs which occupied a basal position to all other members of this genus (Fig. 1).

In addition, two bird species, the Mute Swan and the Mallard, yielded positivity in the PCR detecting acanthamoebae (Table 1). The cloacal samples of four Mute Swans, which were all sampled at the same location (Supplementary Fig. 1), contained four different species: in particular, (1) *Acanthamoeba castellanii*, with 100% (429/429 bp) 18S rRNA gene sequence identity to several isolates of this species reported in GenBank (e.g., AF114438, from the reptile plumed basilisk, *Basilliscus plumifrons* sampled in Austria); (2) a different *Acanthamoeba* species of the same group (T4) with 100% (410/410 bp) sequence identity, among the others, to a human cornea-derived isolate (EF140631); (3) *Acanthamoeba palestinensis* (group T2) that showed 99.8% (410/411 bp) sequence identity to conspecific isolate from hot spring water sampled in Taiwan (GU597009); and (4) a novel *Acanthamoeba* genotype of the T13 group, as indicated by its low maximum identity, only 93.52% (404/432 bp) to a soil isolate from Italy (KF928948) among any other reported variants in GenBank. In addition, the single positive Mallard also harbored an *Acanthamoeba* sp. isolate of the T4 group, having 100% (421/421 bp) identity to an isolate from water in Iran (MT613705).

**Fig. 2:**
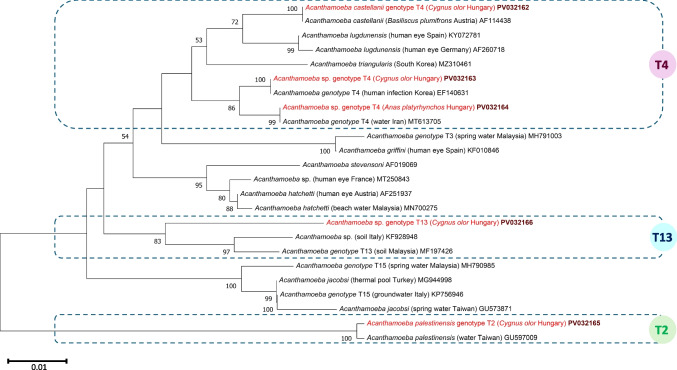
Phylogenetic tree of acanthamoebae based on the 18S rRNA gene. Evolutionary history was inferred using the neighbor-joining method. The percentage of replicate trees in which the associated taxa clustered together in the bootstrap test (1000 replicates) is shown below the branches. The tree is drawn to scale, with branch lengths in the same units as those of the evolutionary distances used to infer the phylogenetic tree. The evolutionary distances were computed using the p-distance method and are in the units of the number of base differences per site. In each row, after the species or genus name, the isolation source and the country of origin (if reported) are shown in parentheses, followed by the GenBank accession number. Sequences from Hungary are indicated with red fonts and maroon, bold accession numbers. The analyses involved 24 sequences and 423 positions. Evolutionary analyses were conducted in MEGA11

The phylogenetic relationships of these avian *Acanthamoeba* isolates confirmed that they belong to five different species (Fig. 2). All *Acanthamoeba*-positive samples were collected in the autumn (Table 1). This implied a significant difference between Mute Swans sampled in the autumn *vs* winter and spring (*P* = 0.025).

In summary, identical genotypes of trichomonads were only found in the same (a single) host species, even in multiple cases (maximum *n* = 7). Within the same geographical location, no intraspecific genetic heterogeneity of *T. gallinarum* was detected, because each of its sequence variants originated at different geographical locations, except two Mallards sampled at the same place but carrying identical genotype (PV032157). By contrast, all five detected *Acanthamoeba* variants (probably belonging to five different species) that were shown to be present in two host species were represented by a single genotype per species, which occurred in the same location (Lake Ady: Supplementary Fig. 1).

## Discussion

In this study, the presence of trichomonads and acanthamoebae was surveyed in a broad range of water-associated bird species, based on molecular analyses of cloacal swabs. The results showed PCR-positivity for trichomonads (mostly *T. gallinarum*) in two synanthropic bird species, the Mallard and the Mute Swan, as well as in Ruffs, but none of the remaining 18 bird species including gulls. This is partly in contrast to a previous report based on similar sampling methods and target birds from Western Europe (The Netherlands), where several gull species but none of the Mute Swans were found infected with trichomonads (Landman et al. 2021). In addition, members of three *Acanthamoeba* genogroups were shown to be present only in two synanthropic waterfowl species, the Mute Swan and the Mallard. Last but not least, in the current study, the DNA of *H. meleagridis* was not found in samples of waterfowl, although this protozoan parasite is present in the study region in domestic fowl (Szekeres et al. 2025).

Considering trichomonads detected in this study, identical genotypes of *T. gallinarum* were found in three Mallards and two other variants (one repeatedly) in Mute Swans. To our knowledge, the clinicopathological manifestation of *Tetratrichomonas* infection among swans was only reported in Black Swan previously (Feng et al. 2021) but not in Mute Swans. The significance of various waterfowl in carrying this protozoan parasite lies in epidemiological risks originating from the presence of infected wild living birds in the area where domestic fowl are kept. In particular, it was hypothesized that wetland birds could be the source of the infection when severe granulomatosis caused by *T. gallinarum* affected productive layer chickens at farms situated in a wetland area in the Netherlands (Landman et al. 2021). In the latter study, three trichomonad species were found: *T. gallinarum*, *Trichomonas tenax*, and *Simplicimonas* sp., of which *T. gallinarum* dominated. Increasing the significance of hosts that carry the latter species in synanthropic environments, several recent studies have identified *Tetratrichomonas* strains isolated from human lungs or the human oral cavity as *T. gallinarum* or *T. gallinarum*-like organisms (Kutisova et al. 2005; Mantini et al. 2009; Lopez-Escamilla et al. 2013; Chen et al. 2024).

In Ruffs sampled in this study, a different *Tetratrichomonas* sp. was detected with high prevalence, which (based on comparison with GenBank sequences and on the results of its phylogenetic analysis) probably represents a new species. Although Ruffs are usually not considered among synanthropic waterfowl in the strictest sense of the word, their presence near domestic animals, e.g., on pastures feeding cattle, is well documented in Hungary: management information from a former study clearly indicates that intensive grazing of grassland (> 1 cow per hectare) attracts a higher abundance of Ruffs (Báldi et al. 2005). In this context, it is noteworthy that the closest relatives of this new species appear to be *Tetratrichomonas* spp. from cattle and pigs (Dufernez et al. 2007; Wylezich et al. 2019).

The third species of trichomonads demonstrated here in a Mallard belonged to the genus *Simplicimonas* and represents a not-yet-described species. Importantly, this variant was hitherto only reported from chickens, including the neighboring Austria (Bilic et al. 2014), emphasizing the potential transmission between these hosts and underlining the epidemiological relevance of the presence of synanthropic waterfowl near domestic poultry. Other examples of a similar connection between humans and domestic animals are also available. For instance, *Simplicimonas similis* DNA was reported persistently from human feces, as well as a zebu (*Bos taurus indicus*) living nearby (Greigert et al. 2018). Literature data also support the potential pathogenic role of chicken-associated *Simplicimonas* sp.-like flagellates (Lollis et al. 2011). Confirming this, a *Simplicimonas* sp. isolate was recently found in association with *T. gallinarum* as the only infectious agent in several layer chickens presenting with granuloma disease in a Dutch farm as well as in a wild duck shot at the affected farm (Landman et al. 2019). Consequently, the same isolate was also found in Black-headed Gulls and Mallards (Landman et al. 2021). These results attested that transfer of trichomonads between wild ducks and flocks of productive layers was possible (Landman et al. 2019).

Members of the genus *Acanthamoeba*, particularly classification to genotypes that pose a higher risk of veterinary-medical implications, are seldom reported from avian hosts, and all published data are available from terrestrial birds. Genotype T4 acanthamoebae were documented in the liver of tucan (Visvesvara et al. 2007), on the oropharyngeal mucosa of captive bustards (Silvanose et al. 1998) and the cornea of wild living predatory birds where they caused keratitis (Karakavuk et al. 2017).

These few literature data attest that acanthamoebae can be present in terrestrial birds, sometimes even infecting their viscera. However, in such cases, there is no exit from the host, and these do not imply contamination of the environment with acanthamoebae. On the other hand, as shown here, their presence in the cloaca of water-associated bird species may imply passing with the feces as reported in reptiles (Tuska-Szalay et al. 2022) and may also explain or at least can contribute to the presence of acanthamoebae in the organs of freshwater fish (Taylor 1977; Dyková et al. 1999; Im and Shin 2003) where Mute Swans and Mallards live. The presence of potentially pathogenic acanthamoebae in edible freshwater fish, as exemplified by *A. castellanii*, may pose a public health risk (Milanez et al. 2024). The latter species and other members of the human-pathogenic T4, T2, and T13 (Grün et al. 2014; Wang et al. 2023) groups were also found in two synanthropic species of waterfowl during this study. Whether these findings are due to actual infection of the cloaca or have retroperistaltic origin owing to cloacal drinking which was reported in Mallards (Wille et al. 2018) but not in swans remains to be clarified.

Additional potential clinico-pathologic consequence of acanthamoebae in the cloaca include (1) the likely transmission of these opportunistic protozoa to the cornea during preening, and *Acanthamoeba* keratitis may develop as reported in birds of prey (Karakavuk et al. 2017). Furthermore, (2) the presence of acanthamoebae in the cloaca and probably the droppings of waterfowl may pose a risk for infection among other waterfowl, fish, and human beings swimming nearby in lakes, particularly if the latter wear soft contact lenses (Ibrahim et al. 2007). Lastly, (3) a similar phenomenon may explain why contact with bird-contaminated material may result in acanthamoeba keratitis among humans (Syam et al. 2005).

*Acanthamoeba* enhances the persistence of *Campylobacter jejuni*, therefore the presence of the amoeba in broiler house environments may have important implications for the ecology and epidemiology of this bacterium species (Snelling et al. 2008; Baré et al. 2010). Another poultry pathogen of high veterinary importance and zoonotic potential, *Mycobacterium avium*, was also reported to associate with acanthamoebae (Berry et al. 2010). Thus, based on the present results it can be speculated that visits of *Acanthamoeba*-carrier waterfowl in a synanthropic environment may enhance the chances of grazing-infection with these bacteria among free-ranging poultry or ruminants, pigs (*nota bene*, acanthamoebae might be present *ab ovo* in the soil, but dispersal by birds means onto the grass and soil).

In this study, no statistically supported seasonal differences were observed between the number of trichomonad-positive birds which were encountered in the autumn, winter, and spring. By contrast, when tetratrichomonosis affected a geese flock, lower mortality rate was observed in the autumn–winter season owing to the lower survival rate of these parasites in cool/cold water, compared to outbreaks typically occurring in the spring–summer season (Ziomko et al. 1991; Falkowski et al. 2022). On the other hand, acanthamoebae were detected here exclusively in the autumn, which may indicate seasonality in line with results of other studies (Kao et al. 2015). However, this may show significant variation depending on climatic and epidemiological conditions, and therefore it is uncertain to ascribe general importance to this observation because of the complexity of local factors influencing it (variations in rainfall, temperature, etc.). This is further supported by our observation that all *Acanthamoeba*-carrier birds of this study were found at the same location. Moreover, the sequence identity of *A. castellanii* between a bird of this study and a reptile in neighboring Austria (Walochnik et al. 1999), i.e., a country within 50-km distance of the location where all positive samples originated, also suggest common environmental source(s) of the infection.

Finally, it should be noted that the low genetic diversity of *T. gallinarum*, as observed in this study (same genotype consistently found in the same bird species, either at the same or at multiple locations), may reflect predominantly direct host-to-host transmission (Hornok et al. 2025). On the contrary, in line with the absence or negligible role of direct transmission between *Acanthamoeba*-carrier hosts, as these protozoa are opportunistically acquired, in this study, identical *Acanthamoeba* species were not found among birds living at the same location.

For assessing the medical risks associated with environmental sources, acanthamoebae are usually screened and reported from artificial bodies of water, such as swimming pools (Kiss et al. 2014). Results of the present study support that natural water surfaces and swimming may also entail infection with non-thermophilic opportunistic amoebae, and waterfowl should be added to the potential sources of such cases. The above results are also particularly relevant to places where it is common practice to flood pastures with lake water.

## Conclusions

Results of this molecular study suggest that synanthropic waterfowl may harbor a broad spectrum, even yet unknown or undescribed species of hind gut trichomonads and acanthamoebae. Some of these may contaminate the environment and can be potentially acquired by domestic poultry and other vertebrates living nearby. These data support that natural water surfaces and swimming may also entail infection with non-thermophilic opportunistic amoebae, and waterfowl should be added to the potential sources of such cases. This is particularly relevant to places where pastures are flooded with lake water.

## Supplementary Information

Below is the link to the electronic supplementary material.

ESM1 Supplementary Fig. 1. Relief map of Hungary showing sampling sites and indicating where Tetratrichomonas spp. (T), Simplicimonas sp. (S) or Acanthamoeba spp. (A) were identified in cloacal samples of waterfowl. (PDF 966 KB)

## Data Availability

The sequences obtained during this study are deposited in GenBank under the following accession numbers: PV032157-PV032166. All other relevant data are included in the manuscript and the supplementary material or are available upon request by the corresponding author.
