# Supplementary figures and images for: Molecular evidence for trichomonads and acanthamoebae in cloacal samples of synanthropic waterfowl
Source: Parasitol Res. 2025 Jul 2;124(7):76. doi: 10.1007/s00436-025-08522-z (PMC12222302; doi:10.1007/s00436-025-08522-z)

# Carpathian Basin

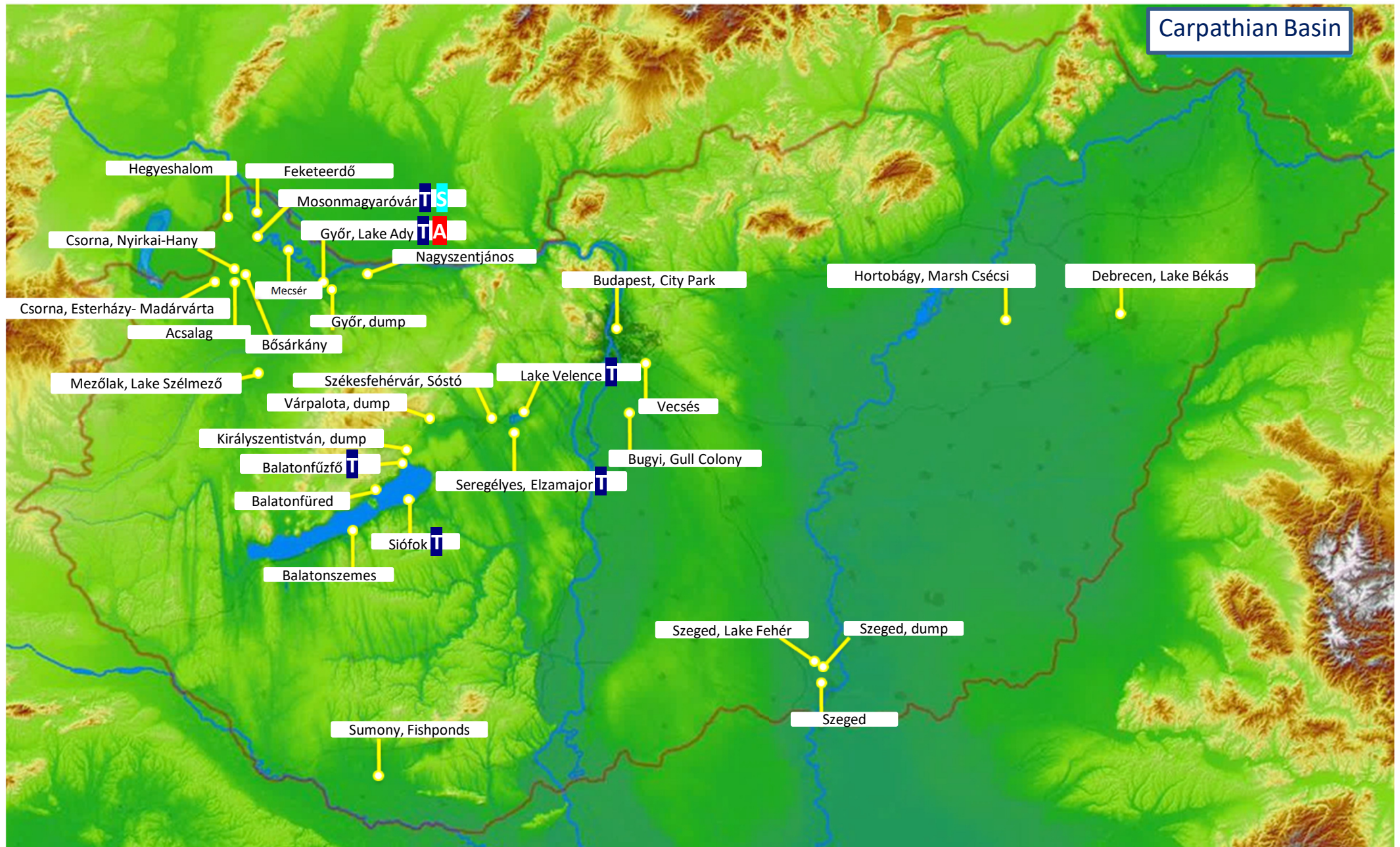

Supplement: Supplementary file 1 — Supplementary Fig. 1. Relief map of Hungary showing sampling sites and indicating where Tetratrichomonas spp. (T), Simplicimonas sp. (S) or Acanthamoeba spp. (A) were identified in cloacal samples of waterfowl. (PDF 966 KB) [file 436_2025_8522_MOESM1_ESM.pdf]
